# Human macrophages and monocyte-derived dendritic cells stimulate the proliferation of endothelial cells through midkine production

**DOI:** 10.1371/journal.pone.0267662

**Published:** 2022-04-27

**Authors:** Elias A. Said, Sumaya Al-Dughaishi, Wadha Al-Hatmi, Iman Al-Reesi, Marwa Al-Riyami, Mohammed S. Al-Balushi, Atika Al-Bimani, Juma Z. Al-Busaidi, Murtadha Al-Khabori, Salam Al-Kindi, Francesco A. Procopio, Afrah Al-Rashdi, Aliyaa Al-Ansari, Hamza Babiker, Crystal Y. Koh, Khalid Al-Naamani, Giuseppe Pantaleo, Ali A. Al-Jabri

**Affiliations:** 1 Department of Microbiology and Immunology, College of Medicine and Health Sciences, Sultan Qaboos University, Muscat, Oman; 2 Department of Pathology, College of Medicine and Health Sciences, Sultan Qaboos University, Muscat, Oman; 3 Department of Hematology, College of Medicine and Health Sciences, Sultan Qaboos University, Muscat, Oman; 4 Laboratory of AIDS Immunopathogenesis, Department of Medicine, Centre Hospitalier Universitaire Vaudois (CHUV) University of Lausanne, Lauzane, Switzerland; 5 Department of Biology, College of Science, Sultan Qaboos University, Muscat, Oman; 6 Department of Biochemistry, College of Medicine and Health Sciences, Sultan Qaboos University, Muscat, Oman; 7 Department of Medicine, Armed Force Hospital, Muscat, Oman; Centro de Investigaciones Biologicas, CSIC, SPAIN

## Abstract

The cytokine midkine (MK) is a growth factor that is involved in different physiological processes including tissue repair, inflammation, the development of different types of cancer and the proliferation of endothelial cells. The production of MK by primary human macrophages and monocyte-derived dendritic cells (MDDCs) was never described. We investigated whether MK is produced by primary human monocytes, macrophages and MDDCs and the capacity of macrophages and MDDCs to modulate the proliferation of endothelial cells through MK production. The TLR stimulation of human monocytes, macrophages and MDDCs induced an average of ≈200-fold increase in MK mRNA and the production of an average of 78.2, 62, 179 pg/ml MK by monocytes, macrophages and MDDCs respectively (*p* < 0.05). MK production was supported by its detection in CD11c^+^ cells, CLEC4C^+^ cells and CD68^+^ cells in biopsies of human tonsils showing reactive lymphoid follicular hyperplasia. JSH-23, which selectively inhibits NF-κB activity, decreased the TLR-induced production of MK in PMBCs, macrophages and MDDCs compared to the control (*p* < 0.05). The inhibition of MK production by macrophages and MDDCs using anti-MK siRNA decreased the capacity of their supernatants to stimulate the proliferation of endothelial cells (*p* = 0.01 and 0.04 respectively). This is the first study demonstrating that the cytokine MK is produced by primary human macrophages and MDDCs upon TLR triggering, and that these cells can stimulate endothelial cell proliferation through MK production. Our results also suggest that NF-κB plays a potential role in the production of MK in macrophages and MDDCs upon TLR stimulation. The production of MK by macrophages and MDDCs and the fact that these cells can enhance the proliferation of endothelial cells by producing MK are novel immunological phenomena that have potentially important therapeutic implications.

## Introduction

Midkine (MK) is a heparin binding growth factor of 13 kDa that is rich in basic amino acids and cysteine. It is a cytokine belonging to the midkine family, which includes only two members, midkine and pleiotrophin [1]. It is a cysteine- and basic- amino acid rich protein consisting of two domains, N- and C- domains, which are flexible regions, linked to each other by a flexible disulfide linker region and each one has three anti-parallel β sheets [2]. The *MDK* gene is located on 11q11.2 chromosome in human [3].

In adults, significant MK expression is observed only in restricted sites e.g. the kidney [4], gut [5], epidermis [6], bronchial epithelium [7] and B-lymphocyte [1]. Although MK was shown to be expressed by myeloid cells in the animal model, only a few studies documented its expression in human myeloid cells. In fact, low levels of MK expression was detected in monocytes in axolotl [8] and its expression was detected in tissue macrophages in rabbits and mice [9, 10]. In human, MK protein expression was induced by hypoxia in neutrophils and monocytes [11]. The expression of MK in macrophage-like cells differentiated from the human monocytic cell line THP-1 is controversial, as Narita et al. did not detect MK in these cells, while Biriken et al. reported that MK is expressed upon the differentiation of THP-1 using PMA [10, 12]. To our knowledge, no study has shown the expression of MK by primary human macrophages and dendritic cells.

MK is involved in development, reproduction, tissue repair, inflammation, innate immunity, control of blood pressure and angiogenesis and it is also strongly expressed during embryogenesis [2]. MK is able to regulate the activity of CD8 and CD4 T cells as well as macrophages, as it interferes with activation of these cells [13]. MK ability to activate CD4 T cells was described [14], however, contradictory effects were reported for CD8 T cells as a study showed an inhibitory effect of MK on CD8 T cells [13] while another study described an activating effect on these cells [15].

MK also plays a role in different diseases including autoimmune diseases such as rheumatoid arthritis, systemic lupus erythematosus, and multiple sclerosis [2, 16–19]. MK elevated levels were associated with poor prognosis in rheumatoid arthritis [20]. Moreover, MK has a role in the differentiation of T helper 1 (Th1) cells that are important in the pathology of systemic lupus erythematosus [14]. MK also suppresses regulatory T cells (T Regs) that are important for the control of multiple sclerosis [18]. MK is also implicated in other diseases like cancer where it enhances the development of different types of cancers by promoting the proliferation of tumor cells [2] and it participates in the resistance to anti-cancer therapy by affecting the activation of CD8 T cells [15]. MK is also induced in many tissues after injury [2] and plays an important role in the healing of injuries by promoting survival and repair as observed in many organs such as the brain, eye, heart and liver [2].

Angiogenesis plays a vital role in cancer and injury healing [21]. The proliferation of endothelial cells is crucial in angiogenesis [2, 3, 22]. MK implication in the induction of endothelial cell proliferation was shown under conditions of addition of soluble MK to these cells [22, 23]. In fact, MK is a proangiogenic factor and its constitutive expression in MCF-7 breast carcinoma cells confers a growth advantage *in vivo*, probably due to its angiogenic activity [23] and its addition to endotehelial cells induced their proliferation [22]. Therefore, MK capacity to promote tumor growth is partially mediated by its capability to enhance angiogenesis in the tumor [3]. Interestingly, macrophages and dendritic cells (DCs) can also promote the proliferation of endothelial cells and are involved in angiogenesis [24, 25]. The implication of MK in the mechanisms by which macrophages and DCs promote angiogenesis [24, 25] is not documented because the production of MK by primary human MDDCs and macrophages was never demonstrated as mentioned above. In addition, the potential production of MK by innate APCs would be of great importance for many diseases and mechanisms in which MK is implicated. Therefore, we investigated if human monocytes-derived DCs (MDDCs) and macrophages, produce MK, as the production of MK was never demonstrated in human MDDCs and macrophages, and the effect of MK produced by these cells on proliferation of endothelial cells.

## Materials and methods

### Study population

Blood was collected from 25 healthy donors (age = 29.8 ± 8 years, F/M ≈1). The number of donors included in each experiment is indicated in the corresponding figure. An informed consent was obtained from all donors.

Formalin fixed paraffin embedded specimens of tonsils and lymph nodes showing reactive lymphoid follicular hyperplasia from 4 patients were obtained from the Pathology Department at the Sultan Qaboos University Hospital (SQUH), Muscat, Oman. The procedure consisted of staining performed on biopsies that were stored in the bank of the Pathology Department, and did not involve any special sample collection (article 32 of the Declaration of Helsinki), patient consent was impracticable to obtain.

The study and the procedure (including the absence of consent for the biopsies) were approved by the Medical Research Ethics Committee of the College of Medicine and Health Sciences in the Sultan Qaboos University (SQU) MERC#1654. The data were analyzed anonymously. To maintain confidentiality, every donor and patient was assigned with a unique identification number.

### Isolation of peripheral blood mononuclear cells (PBMCs) and monocytes and differentiation of macrophages and MDDCs

Blood samples were collected in CPDA-1 bags (Terumo, Japan). PBMCs were isolated from blood samples using Ficoll-Hypaque density gradient technique (Sigma-Aldrich, Germany).

Monocytes were isolated by adherence for 2hrs. in RPMI media with no serum or using Pan Monocyte Isolation kit (Miltenyi Biotec, Germany) according to the manufacturer instructions. Macrophages were obtained from monocytes cultured for 6 days in RPMI media (Gibco, UK) supplemented with 50 ng/ml human recombinant granulocyte-macrophage colony-stimulating factor (GM-CSF; R&D, UK). MDDCs were differentiated from monocytes for 6 days in RPMI media supplemented with 50 ng/ml GM-CSF and 50 ng/ml of human recombinant interleukin-4 (IL-4; R&D, UK). Cells (10^6^ cells/ml) were stimulated for 24 hrs with 20ng/ml lipopolysaccharide (LPS; InvivoGen, USA).

### Isolation of plasmacytoid DCs (pDCs) and myeloid DCs (mDCs)

Plasmacytoid DCs (pDCs) and myeloid DCs (mDCs) were separated from PBMCs by Plasmacytoid Dendritic Cell Isolation kit II, CD1c (BDCA-1)^+^ Dendritic Cell Isolation kit and Myeloid Dendritic Cell Isolation kit (Miltenyi Biotech, Germany) according to the manufacturer’s instructions. DCs (10^6^ cells/ml) were stimulated with lipopolysaccharide (LPS) as stated above, while pDCs were stimulated with 5μg/ml resiquimod (InvivoGen, USA) because they express Toll-like receptors (TLRs) 7 and 9 only. Non-stimulated cells were used as control. Cells (10^6^ cells/ml) were incubated for 24 hours.

### Flow cytometry

Cell activation (CD80 and CD86 upregulation) and purity were assessed by flow cytometry LSRFortessa™ cell analyzer (BD, USA) using monoclonal mouse anti-human Abs anti-CD3 (561805, ID AB_10893800), CD14 (557923, ID AB_396944), CD19 (557921, ID AB_396942), CD56 (557919, ID AB_396940)-Alexa700, HLA-DR-APC-Cy7 (335831, ID AB_2868692), CD123-PE (340545, ID AB_400052), CD11c-APC (559877, ID AB_398680), CD80-PEcy5 (559370, ID AB_397239) and CD86-PEcy7 (561128, ID AB_10563077). The dilutions were according to the manufacturer’s instructions (BD, USA). Upon differentiation, the monocyte-derived macrophages were identified by flow cytometry as CD14^+^ cells and MDDCs as CD14^-^ cells.

### Assessing production of MK mRNA by real time PCR

Total RNA was extracted from cells using PureLink RNA Mini Kit (ambion,life technologies,USA) according to the manufacturer’s instructions. The concentrations of RNA samples were determined by NanoDrop technology. The RNA samples were treated with RQ1 RNase-Free DNase (Promega kit, USA). Complementary DNA (cDNA) was synthesized from the RNA samples using a reverse transcriptase enzyme (Thermo Scientific, USA). Relative quantitative real-time PCR was performed using GoTaq® qPCR Master Mix kit (Promega, USA) and 7500 Fast Real-Time PCR (Applied Biosystems, US). MK forward primers: 5^’^-CGACTGCAAGTTTGAGAAC-3’ and reverse primers: 5^’^-TCTCCTGGCACTGAGCATTG-3^’^. β-tubulin was used as housekeeping gene for normalization. Its forward primers: 5^’^-AAG GAG GTC GAT GAG CAG AT-3^’^ and reverse primers: 5^’^-GCT GTC TTG ACA TTG TTG GG-3^’^. The PCR parameters were 1 cycle of 95°C for 2 minutes (for polymerase activation), followed by 35 cycles of 95°C for 15 seconds and 60°C for 1 minute. Expression was measured by comparing ΔCt between target gene and the reference gene of the same sample. Fold-differences were then calculated using the following formula 2^-ΔΔCT^.

### Assessing MK and TNF-α protein production

The MK protein was detected in the supernatant using MK human ELISA kit (Biovendor, Germany) according to the manufacturer’s instructions. TNF-α was detected in the supernatant using Cytometric Bead Array (CBA; BD, USA).

### Immunohistochemistry (IHC) and identification of cells

The sections were stained using the Ventana BenchMark Ultra Slide Staining system (Roche, Switzerland), with the use of the UltraView Universal DAB and UltraView Universal AP RED Detection kits (Roche, Switzerland). The procedure consisted of heating at 72°C for 8 min., then at 97°C under a high pH for 64 min. The incubation with the mouse anti-human monoclonal anti-MK (sc-46701, ID AB_627949) antibody (Santa Cruz, USA) at dilutions 1/25, 1/50 or 1/100 for 2 hrs. was followed by a 40 min. incubation with rabbit anti-human monoclonal anti-CD11c (ab52632, ID AB_2129793), anti-CLEC4C (ab239077, ID AB_2904588) or anti-CD68 (ab213363, ID AB_2801637) antibodies (Abcam, UK). The dilutions of these antibodies were according to the manufacturer’s instructions. Counterstaining with hematoxylin was performed during 16 min. Positive cells were observed in at least five high power fields (diameter of the field = 0.44 mm) with 60x objective and 10x ocular lenses (Olympus BX53). Positive cell identification was done by four independent expert/trained readers.

### Electroporation of cells in the presence of siRNA

Cells (10^6^ cells/ml in 2 mm gap cuvette) were electroporated (200 Volts, 2 pulses and 500 μsec/pulse) with BTX ECM 830 square-wave electroporator (BTX Harvard Apparatus- PR), in the presence of 600 nM control-siRNA or MK-siRNA containing a mixture of siRNA targeting MK mRNA sequence (Sigma Aldrich, Germany), then incubated for 16 hrs and stimulated by LPS for 24 hrs. The supernatant was collected after the incubation period to test MK production by ELISA.

### Cell proliferation

Human umbilical vein endothelial cells (HUVEC; material number 200-05N, Thermo Fisher, USA, received in October 2019) were cultured according to the manufacturer instructions. Cells were carboxyfluorescein succinimidyl ester (CFSE; E-Bioscience, USA)-labelled and incubated (10^5^cells/well, 4 days) in the presence or not of macrophage or MDDC supernatants and soluble MK (Biovendor, Germany) as indicated. HUVEC proliferation was monitored using flow cytometry. CFSE covalently binds to free amine residues after it passively diffuses into the cells. After cellular division, the amount of CFSE in each daughter cell is the half of that present in the mother cell. Therefore, CFSE MFI in the cells decreases when cells proliferate [26]. To assess cell proliferation, we calculated the percentage, by which the MFI of the CFSE-labelled cells decreased in the supernatant-treated samples compared to the MFI of the CFSE-labelled cells that were incubated with the medium alone.

### Assessing cell viability

Cells were stained with Annexin-V-PE (BD, USA) and 7AAD (BD, USA) according to the manufacturer’s instructions. Cells were monitored using flow cytometry.

### Statistical analysis

To assess the significance of the observed differences between two variables due to the effect of treatments on the cells, we used 2-tailed paired t-test. A *p* value < 0.05 was considered as significant. The error bars indicate the standard deviation in all figures. Microsoft Excel was used for statistical analysis.

## Results

### MK production by human monocytes and their derived macrophages and MDDCs

**Fig 1:**
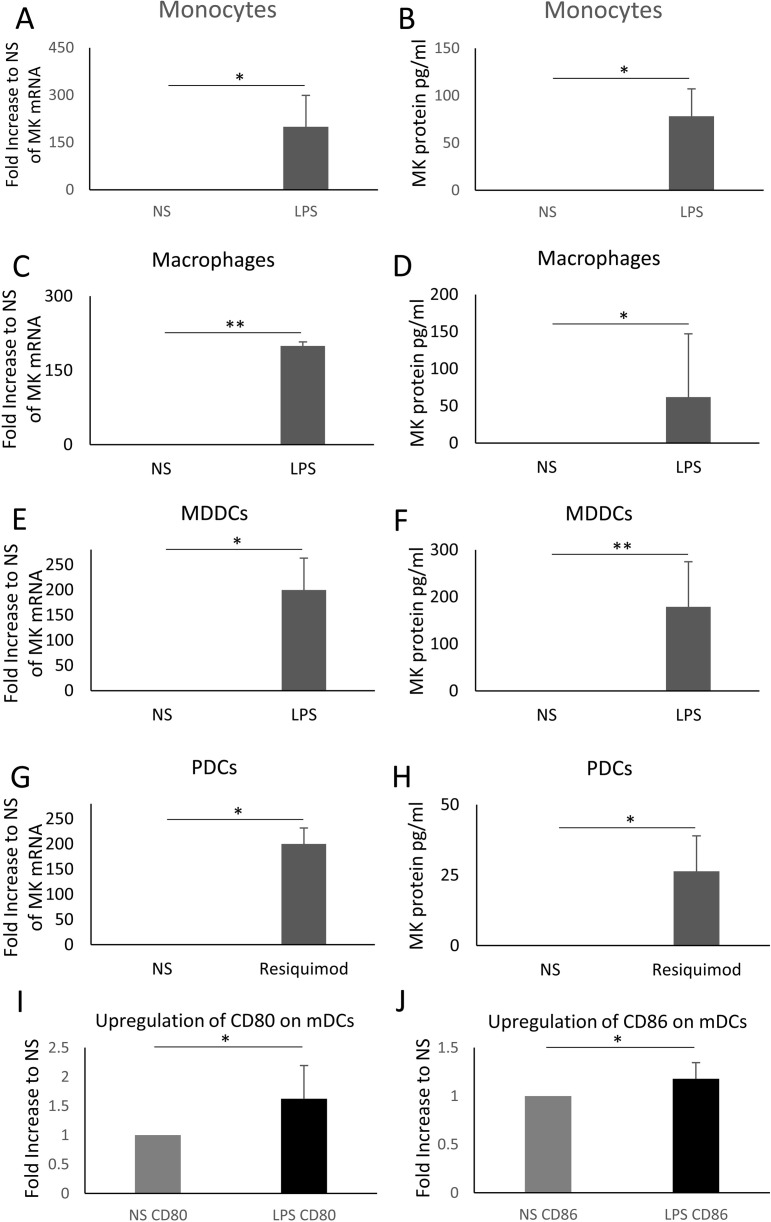
MK production by human monocytes, macrophages, MDDCs and pDCs. Monocytes (n = 3; A and B), macrophages (n = 4; C and n = 5; D) and MDDCs (n = 4; E and F), obtained from human PBMCs, were stimulated with LPS for 24 hrs (10^6^ cells/ml). PDCs, isolated from human PBMCs, were stimulated with resiquimod (n = 3; G and H). MK mRNA in the cell lysates and protein in the supernatant were detected by real time PCR and ELISA, respectively. The upregulation of CD80 (I) and CD86 (J) on the surface of mDCs treated with LPS. NS = Non stimulated. N = number of donors. A 2-tailed paired t-test was applied to assess the significance of the observed differences * *p* < 0.05, ** *p* < 0.01. The error bars indicate the standard deviation.

We investigated the production of MK mRNA and protein by human monocytes and their derived macrophages and DCs. The stimulation of monocytes (n = 3) with 20ng/ml LPS induced ≈200 fold-increase in the levels of MK mRNA, *p* = 0.049 (Fig 1A) and an average of 78.2 pg/ml (ranged from 57.5 to 88.7 pg/ml, *p* = 0.021) of MK protein (Fig 1B). A similar increase was observed in macrophages (n = 4) stimulated with 20ng/ml LPS [≈200 fold-increase in MK mRNA, *p* = 0.008 and an average of 62 pg/ml (n = 5; ranged from 18 to 226.4 pg/ml, *p* = 0.049) of MK protein (Fig 1C and 1D)], and MDDCs [n = 4, ≈200 fold-increase in MK mRNA, *p* = 0.042 and an average of 179 pg/ml (ranged from 23 to 222.7 pg/ml, *p* = 0.002) of MK protein (Fig 1E and 1F)].

To compare with circulating DCs we investigated the production of MK by pDCs and mDCs. Because human pDCs do not express TLR4 [27, 28], we used resiquimod, an effective analogue of imiquimod that stimulates TLRs 7 and 8 [29], to stimulate these cells instead of the TLR4 ligand LPS. The stimulation of pDCs (n = 3) with 5μg/ml resiquimod induced ≈200 fold-increase in the levels of MK mRNA, *p* = 0.028 (Fig 1G) and an average of 26.4 pg/ml (*p* = 0.049; range from 15.5 to 39.9 pg/ml) of MK protein (Fig 1H). We did not detect MK production in LPS-stimulated mDCs isolated by direct (n = 3) and indirect (n = 3) binding to magnetic beads (n = 3 for each) although these cells upregulated CD80 and CD86 on their surface upon stimulation as observed by flow cytometry (Fig 1I and 1J).

**Fig 2:**
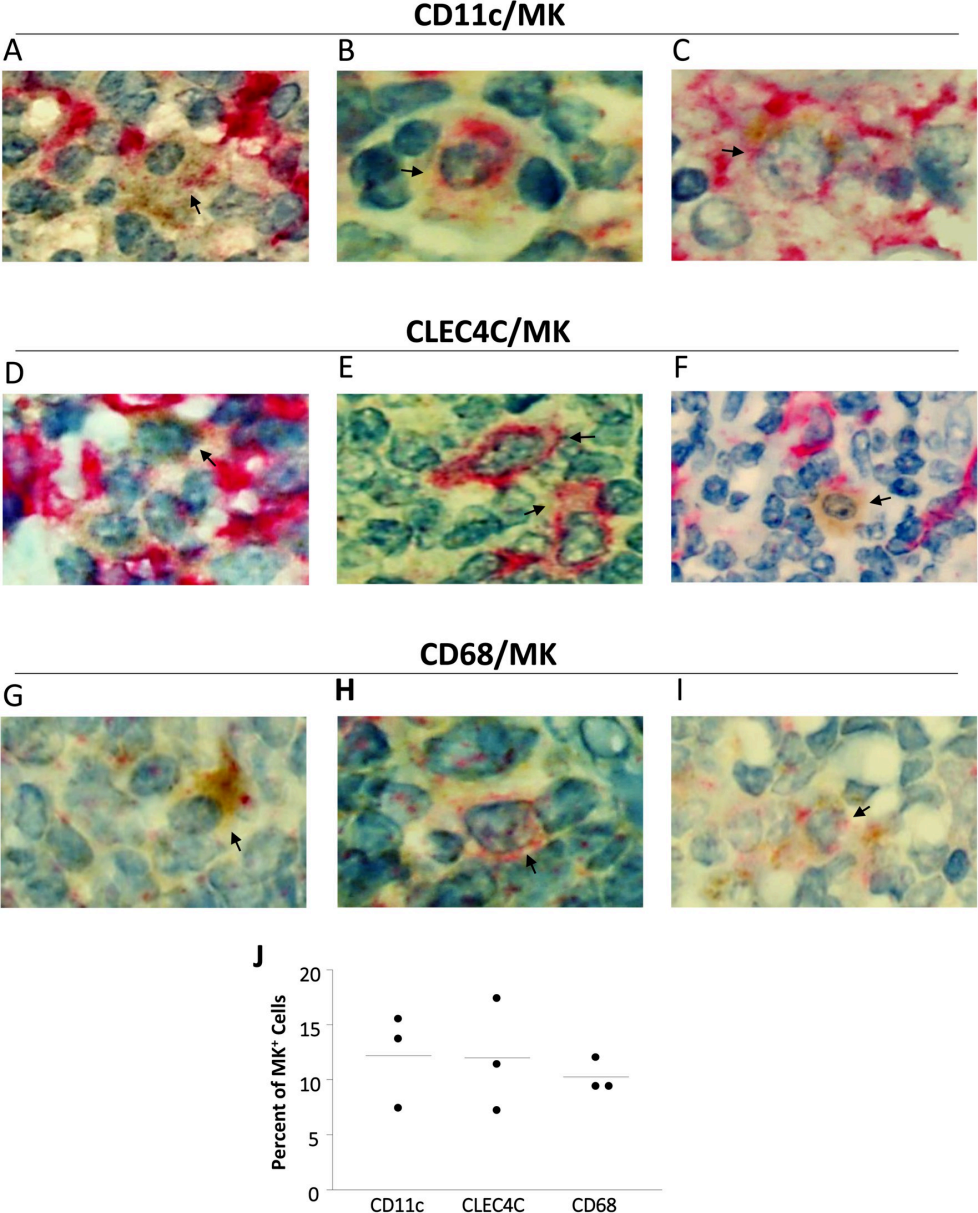
MK is produced by human CD11c^+^, CLEC4C^+^ and CD68^+^ cells in the tonsils. Sections of tonsils showing reactive lymphoid follicular hyperplasia were stained with anti-MK antibody and anti-CD11c, anti-CLEC4C or anti-CD68 antibodies. Representative pictures of MK staining (brown color) detected in CD11c^+^ (red color) cells (A-C), CLEC4C^+^ (red color) cells (D-F) and CD68^+^ (red color) cells (G-I) in tonsils showing reactive lymphoid follicular hyperplasia (n = 3). Cells producing MK are indicated by arrows in the pictures. MK staining appears as brown and CD11c, CD68 and CLEC4C staining appears as red. The percentage of MK^+^CD11c^+^, MK^+^CLEC4C^+^ and MK^+^CD68^+^ cells to the total CD11c^+^, CLEC4C^+^ and CD68^+^ cells respectively (J). N = number of donors.

Our results demonstrated that MK is expressed by human MDDCs, pDCs, monocytes and macrophages. Therefore, as a proof of concept, we investigated if MK production can be detected in these cells *in vivo*. For this purpose, we performed double staining IHC using Abs specific of MK and markers of different cell subsets. Both CD11c and CD1c identify MDDCs [30], and CD11c can identify DCs that differentiate from monocytes *in vivo* [31]. However, both of these markers are also expressed by other cell types such as myeloid classical DC2 (cDC2), macrophages, monocytes, a subset of B cells and granulocytes [30, 32, 33]. Therefore, we used antibodies directed against CD11c to co-stain with the anti-MK Ab and we referred to the cells expressing CD11c as CD11c^+^ cells. To identify pDCs we used antibodies directed against CLEC4C (CD303) that is specifically expressed by pDCs [30, 34] and we refer to the cells expressing CLEC4C as CLEC4C^+^ cells. Moreover, monocytes and macrophages can be identified using antibodies directed against CD68, CD163, CD14 and CD16. However, all these markers can be expressed by subsets of DCs as well [30, 35]. Moreover, because CD163, CD14 and CD16 levels can be modulated by stimulation [36], we used antibodies directed against CD68 [36, 37]. Interestingly, CD68 is expressed in monocytes, macrophages and subsets of DCs in the tonsils [35], which supports our choice of this marker. We refer to the cells expressing CD68 as CD68^+^ cells. We stained sections of tonsils showing reactive lymphoid follicular hyperplasia (n = 3) with anti-MK Ab and anti-CD11c, anti-CD68 or anti-CLEC4C Abs. We have observed MK production in CD11c^+^ cells (Fig 2A–2C), in CLEC4C^+^ cells (Fig 2D–2F) and CD68^+^ cells (Fig 2G–2I). We calculated the percentage of MK^+^CD11c^+^, MK^+^CLEC4C^+^ and MK^+^CD68^+^ cells to the total CD11c^+^, CLEC4C^+^ and CD68^+^ cells respectively by counting these cells in 6 different fields for each sample. The average percentage of MK^+^CD11c^+^ cells was 12.2% (ranging from 7.4% to 15.5%), MK^+^CLEC4C^+^ cells was 12% (ranging from 7.2% to 17.4%) and MK^+^CD68^+^ cells was 10.3% (ranging from 9.4% to 12%; Fig 2J).

**Fig 3:**
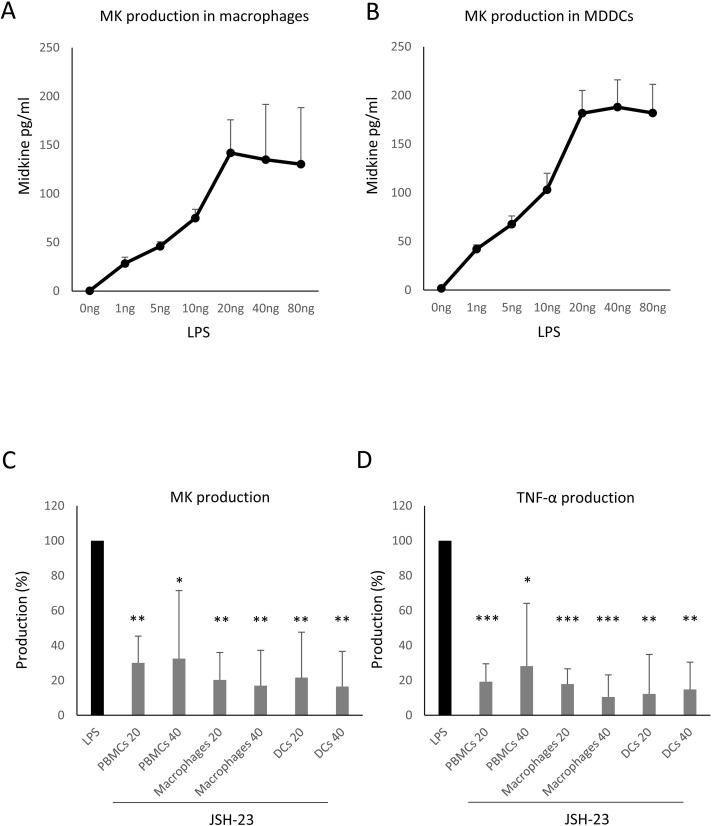
NF-κB plays a potential role in the TLR-induced MK production in macrophages and MDDCs. Macrophages (A) and MDDCs (B) were treated with the indicated LPS concentrations for 24 hrs. MK was detected in the supernatant by ELISA (n = 3). Human PBMCs, macrophages and MDDCs (10^6^ cells/ml; n = 4) were pre-treated or not with JSH-23 for 1hr. Cells were then stimulated with LPS (20 or 40 ng/ml) in the presence or not of 10 μM JSH-23 for 24 hrs. **C.** MK was detected in the supernatant by ELISA and **D.** TNF-α was detected in the supernatant by CBA. N = number of donors. A 2-tailed paired t-test was applied to assess the significance of the observed differences * *p* < 0.05, ** *p* < 0.01, *** *p* < 0.001 compared to cells treated with LPS alone. The error bars indicate the standard deviation.

We also investigated the induction of MK production upon stimulation of macrophages and MDDCs with LPS in a dose dependent manner. Both cells produced MK upon stimulation with 1 ng/ml LPS (an average of 28.3 pg/ml of MK produced by macrophages and 42 of MK produced by MDDCS; Fig 3A and 3B). MK production reached saturation in both cell types when stimulated with 20 pg/ml LPS (an average of 142 pg/ml of MK produced by macrophages and 181.7 of MK produced by MDDCS; Fig 3A and 3B).

### NF-κB plays a potential role in MK production by human macrophages and MDDCs upon TLR triggering

NF-κB has been shown to be implicated in MK production in prostate cancer cell lines [38] and TLR stimulation induces the activation of NF-κB [39]. Therefore, we investigated whether NF-κB has a role in MK production upon TLR triggering in macrophages and dendritic cells. This was done by assessing the effect of 5 μM JSH-23 (Sigma-Aldrich, Germany), a selective inhibitor of NF-κB [40, 41] that inhibits its activity by blocking NF-κB p65 translocation [42, 43], on the production of MK upon PBMCs, macrophages and MDDCs (n = 4 for each) triggering with 20 and 40 ng/ml LPS. The treatment of PBMCs with JSH-23 decreased the production of MK to 30% and 32.5%, induced with 20 and 40 ng/ml of LPS, respectively, compared to that induced by LPS (*p* = 0.0028 and 0.018, respectively; Fig 3C). Such treatment decreased the production of MK by macrophages to 20.27% and 17%, induced with 20 and 40 ng/ml of LPS, respectively, compared to that induced by LPS (*p* = 0.002 and 0.004, respectively; Fig 3C). The treatment with JSH-23 also decreased the production of MK by MDDCs to 21.75% and 16.5%, induced with 20 and 40 ng/ml of LPS, respectively, compared to that induced by LPS (*p* = 0.009 and 0.004, respectively; Fig 3C). This suggests that NF-κB plays a role in the production of MK upon TLR triggering.

NF-κB is involved in the production of TNF-α upon TLR triggering [44] and the NF-κB inhibitor, JSH-23, blocks TNF-α production induced by LPS [43]. Therefore, we have investigated whether TNF-α production was affected as a control of JSH-23 activity. The treatment of PBMCs with JSH-23 decreased the production of TNF-α to 19.25% and 28.1%, induced with 20 and 40 ng/ml of LPS, respectively, compared to that induced by LPS (*p* = 0.005 and 0.011, respectively; Fig 3D). Such treatment decreased the production of TNF-α by macrophages to 17.8% and 10.5%, induced with 20 and 40 ng/ml of LPS, respectively, compared to that induced by LPS (*p* = 0.0003 and 0.0008, respectively; Fig 3D). The treatment with JSH-23 also decreased the production of TNF-α by MDDCs to 12.2% and 14.8%, induced with 20 and 40 ng/ml of LPS, respectively, compared to that induced by LPS (*p* = 0.0045 and 0.0017, respectively; Fig 3D).

### MK produced by MDDCS and macrophages stimulates the proliferation of human vascular endothelial cells

Monocytes have a short half-life when they are circulating in the blood [45] and stimulating them through their TLRs might lead to undesirable immune responses [46]. They differentiate in the tissues to macrophages or MDDCs [47] that can modulate endothelial cells proliferation [48]. Moreover, our results demonstrated that macrophages and MDDCs could produce MK, and as mentioned above MK is able to stimulate the proliferation of vascular endothelial cells (VECs) [22, 23]. Therefore, we investigated the capacity of MDDCs and macrophages to promote the proliferation of endothelial cells through MK production. For this purpose, we used HUVECs, which are a physiological representative of human VECs [49].

**Fig 4:**
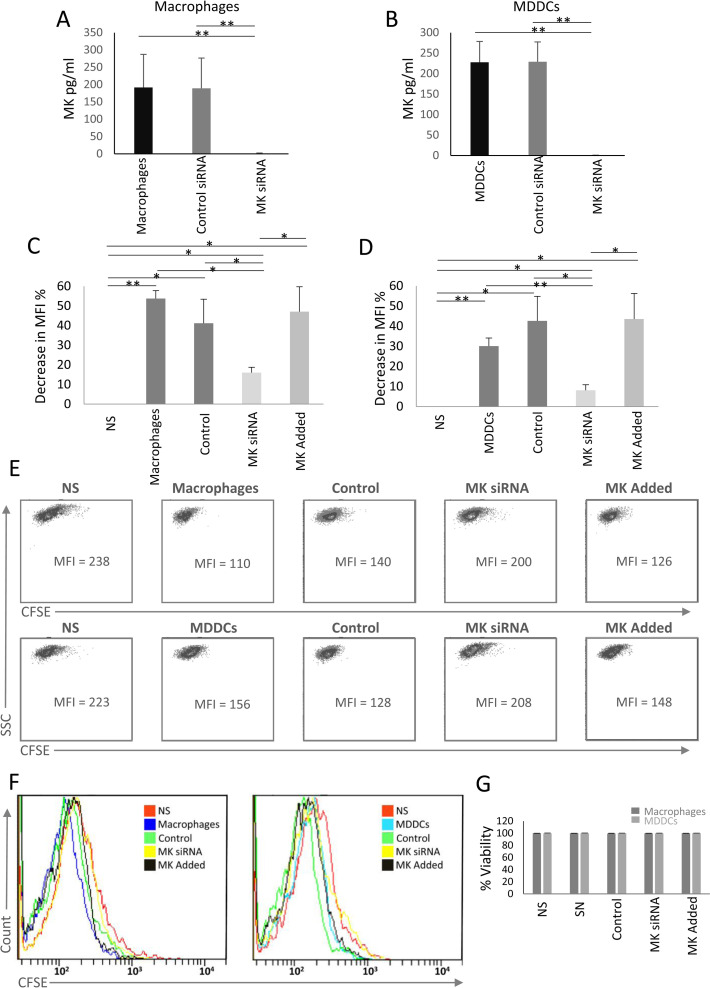
Macrophages and MDDCs stimulate the proliferation of HUVECs through MK production. Macrophages and MDDCs were electroporated with control or MK-specific siRNAs (n = 3). Cells (10^6^ cells/ml) were stimulated with LPS for 24 hrs. MK levels in the supernatants of **A.** macrophages and **B.** MDDCs were measured by ELISA. CFSE-labelled HUVECs (10^5^ cells/well) were incubated for 4 days in the presence of the supernatants of stimulated **C.** macrophages or **D.** MDDCs non-electroporated (indicated as DC and macrophage) or electroporated with the control siRNA or MK siRNA (n = 3). Soluble human MK (200 pg/ml) was added to HUVECs incubated in the presence of the supernatants of macrophages or MDDCs electroporated with MK-specific siRNA (MK Added). **E.** Representative flow cytometry plots showing the CFSE labeling and its MFI in HUVECs incubated as indicated above **F.** Representative flow cytometry histograms showing the CFSE labeling in HUVECs incubated as indicated above. **G.** The percentages of annexin V and 7AAD negative HUVECs incubated under the conditions mentioned above as observed by flow cytometry. NS = Non stimulated (no supernatant). A 2-tailed paired t-test was applied to assess the significance of the observed differences ** *p* < 0.01, * *p* < 0.05. N = number of donors. The error bars indicate the standard deviation.

MDDCs and macrophages (10^6^ cells/ml, n = 3 for each) were electroporated or not with MK-specific or control siRNAs. The electroporation of these cells with MK-specific siRNA inhibited 100% of the production of MK upon stimulation with LPS, while the cells electroporated with the control siRNA produced MK [average ≈ 189.3 pg/ml for macrophages and ≈ 229 pg/ml for MDDCs (Fig 4A and 4B)]. The incubation of HUVECs in the presence of the supernatant of LPS-stimulated MDDCs and macrophages electroporated or not with the control siRNA stimulated the proliferation of HUVECs. This was observed by flow cytometry by the decrease in the MFI of CFSE-labelled HUVECs incubated in the presence of these supernatants, compared to those incubated with the medium alone [an average of MFI decrease ≈ 41.1% and 53.7% for supernatants of macrophages electroporated or not with the control siRNA respectively; *p* = 0.046 and 0.005 respectively; (Fig 4C) and an average of MFI decrease ≈ 42.6% and 30% for supernatants of MDDCs electroporated or not with the control siRNA respectively; *p* = 0.041 and 0.002 respectively; (Fig 4D)]. This decrease was lower in HUVECs incubated in the presence of the supernatants of macrophages or MDDCs electroporated with MK-specific siRNA [(15.9% and 8.1% respectively; *p* = 0.047 and 0.028 respectively; (Fig 4C–4F)]. The addition of soluble MK, at concentrations similar to those produced by macrophages and MDDCs, to HUVECs incubated in the presence of the supernatants of macrophages or MDDCs, electroporated with MK-specific siRNA, induced a decrease in the MFI of CFSE-labelled HUVECs to levels similar to those observed with the cells incubated with the supernatants of macrophages and MDDCs electroporated or not with the control siRNA (Fig 4C–4F). This showed that the inhibition of MK production by these cells decreased the capacity of MDDCs and macrophages to stimulate HUVECs proliferation.

We did not observe any effect on HUVEC viability when these cells were incubated with the supernatants of macrophages or MDDCs electroporated with MK-specific siRNA (Fig 4G). Indicating that the absence of MK did not affect the viability of HUVECs.

**Fig 5:**
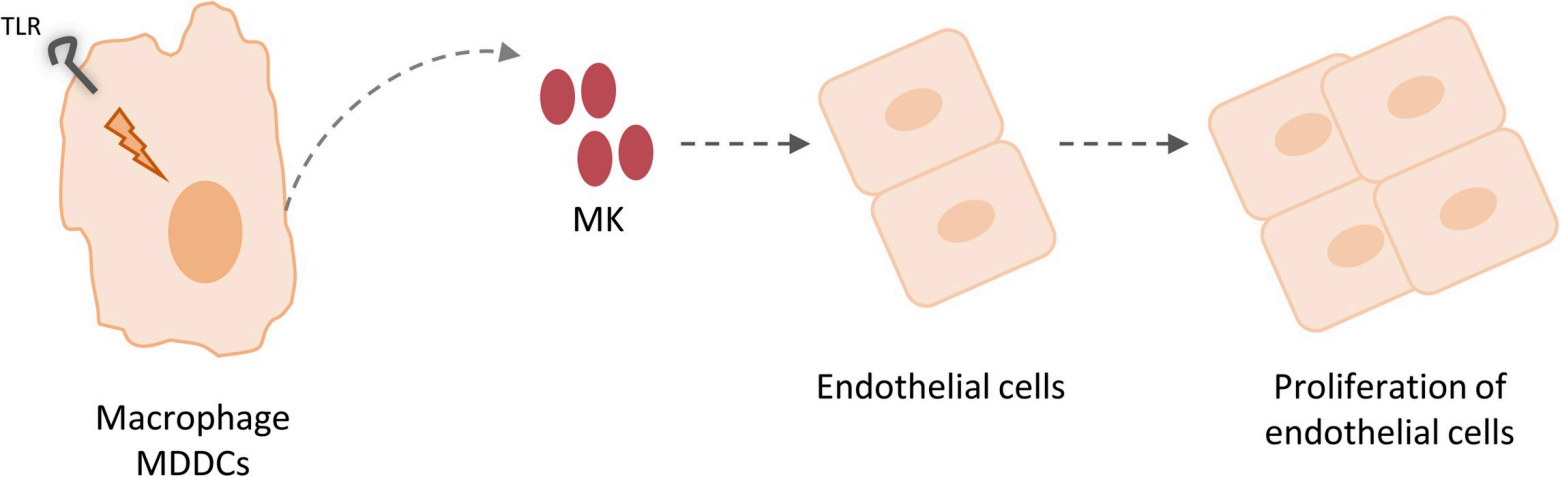
Schematic representation of the capacity of macrophages and MDDCs to stimulate the proliferation of endothelial cells through MK production.

Our results suggest that macrophages and MDDCs stimulate the proliferation of endothelial cells through MK production (Fig 5).

## Discussion

This is the first study to demonstrate the production of MK by primary human monocytes, macrophages and MDDCs. The levels of MK produced by monocytes, macrophages, MDDCs and pDCs were comparable to those produced by the non-stimulated human monocytic cell line THP-1 cultured for 24 hrs and ≈8- to 55.2-fold lower than those produced by PMA-stimulated THP-1 cells [12]. This difference might be related to the fact that THP-1 cells are leukemic cell lines and that their activation profile and physiology are not similar to primary human monocytes and other innate APCs [50]. Of note, MK levels detected in our study are comparable to IL-12 and IL-10 levels produced by monocytes, mDCs and pDCs upon TLR stimulation [51]. The fact that malignant cells can produce higher levels of MK is supported by the fact that levels of MK produced by monocytes, macrophages, MDDCs and pDCs, as observed in our study, are also lower than those produced by tumor cells. They are ≈3.5- to 25.8-fold lower than those produced by human A549 cells (lung squamous cell carcinoma), ≈4- to 27.6-fold lower than those produced by human U87 cells (glioma) and ≈45- to 310-fold lower than those produced by human WM164 (melanoma) cells [15]. The normal range of MK levels in the blood of healthy individuals is not precisely documented. Different studies have reported variable levels of MK in the blood of healthy individuals. The reported average values ranged between 65.6 ± 14.76 pg/mL and 2870 ± 100 pg/ml [52–60], with a few individuals having levels of 0 pg/ml of MK in their blood [52]. However, these studies agree on the presence of a constant production of MK in healthy individuals [52]. The detected levels of MK in our study suggest that monocytes, macrophages, MDDCs and pDCs might participate in the production of MK in healthy individuals, however in the absence of strong inflammatory conditions that lead to the robust stimulation of these innate APCs, other cells might constitute the major source of MK in healthy individuals. The levels of MK in the blood increase in obesity and different inflammatory conditions such as systemic Lupus erythematosus (SLE), rheumatoid arthritis, Crohn’s disease and sepsis, as well as different types of cancer such as esophageal, gastric, duodenal, colon, hepatocellular, bile-duct and gallbladder, pancreatic, thyroid and lung carcinomas [52–56, 58–62]. It is very tempting to hypothesize that MK produced by monocytes, macrophages, MDDCs and pDCs, participates in increasing MK levels in the blood, as these cells play important roles in the inflammatory diseases and cancers [63].

In our study, MK production was induced by TLR triggering. This indicates that MK can be produced by these cells in conditions that involve the presence of TLR ligands including pathogen-associated molecular patterns (PAMPs) in infections and damage-associated molecular patterns (DAMPs) in injured tissues and tumors [64]. The production of MK is not general or specific to myeloid innate antigen presenting cells as this production is not detected in mDCs while pDCs produced MK. Accordingly, we have detected the production of MK by CD11c^+^ cells, CLEC4C^+^ cells and CD68^+^ cells in tissues from human tonsils showing reactive lymphoid follicular hyperplasia. Although CD11c might be the best choice to identify DCs that differentiate from monocytes *in vivo* [31], it is also expressed by other cell types as mentioned above [30, 32, 33]. Therefore, the exact identity of the CD11c^+^ cells producing MK remains to be elucidated. CLEC4C is specifically expressed by pDCs [30, 34], which suggests that pDCs in the tonsils can produce MK. CD68 is expressed in monocytes, macrophages and subsets of DCs in the tonsils [35], suggesting that monocytes, macrophages and/or DCs in the tonsils can also produce MK. The fact that CD68 and CD11c can be expressed together on monocytes, macrophages and DCs in the tonsils [35], suggests that at least a part of the CD11c^+^ MK producing cells might be CD68^+^ cells, which can be monocytes, macrophages and/or DCs. This indicates that MK production by DCs and macrophages should be investigated in infections, tumors and injuries that lead to the activation of these cells [64].

Our results showed that the selective inhibitor of NF-κB [40, 41], JSH-23, which inhibits its activity by blocking NF-κB p65 translocation [42, 43], inhibited MK production by macrophages and MDDCs upon stimulation with LPS. JSH-23, blocks inflammatory cytokine production induced by LPS [43] and it was used to study effects of NF-kB inhibition on biological activities [41, 42, 65, 66]. NF-kB inhibitors are used to demonstrate its implication in the production of different molecules including MK [38, 40, 65, 67]. Therefore, our results suggest that NF-κB plays a potential role in MK production upon TLR stimulation in macrophages and MDDCs. This is supported by the fact that MK gene can be induced by this nuclear factor as shown in cell lines [38], the presence of a putative NF-κB-binding site in the 5’ non-coding region of the MDK gene [68] and the fact that TLR triggering leads to the activation of NF-κB [39].

Moreover, our results showed that macrophages and MDDCs were able to stimulate the proliferation of HUVECs, which constitutes a physiological representative of human vein endothelial cells (VECs) [49], through MK production. This is supported by the fact that soluble MK stimulated the proliferation of these cells [22, 23] and that DCs and macrophages can activate the proliferation of endothelial cells [24, 25]. This shows that MK produced by macrophages and DCs plays a role in endothelial cell proliferation. This is potentially important in angiogenesis a process that is crucial for tissue repair and for the survival of tumor cells [69]. Therefore, interfering with the production of MK by antigen presenting cells (APCs) by negatively regulating its production to limit angiogenesis in tumors and by inducing this production to stimulate angiogenesis for tissue repair represents a potentially important therapeutic strategy. Other factors that are produced by MDDCs and macrophages might also be implicated in the stimulation of endothelial cell proliferation, as the inhibition of this stimulation upon inhibiting MK production was not complete as shown by our results. Of note, the presence or absence of MK in the supernatant of macrophages and MDDCs did not affect the survival of HUVECs as observed by our results.

Macrophages and DCs have an important influence on the cells of the adaptive immune system. Among their effects on B cells, macrophages and DCs, is their capacity to increase the survival of these cells [70, 71]. Taking into consideration that MK induces a signaling cascade that lead to the survival of B cells [1], it would be interesting to investigate whether MK plays a role in the capacity of macrophages and DCs to regulate B cell survival. Different effects were also described for MK on T cells. MK was shown to activate CD4 T cells and have a role in the differentiation to T helper 1 (Th1) cells [14]. This corroborates with the fact that MK was also shown to have an inhibitory effect on the differentiation of T Regs [72]. However, contradictory effects were reported for MK on CD8 T cells. A study showed that MK can have an activating effect on CD8 T cells [13], while another study reported that MK indirectly promotes CD8 T cell dysfunction [15]. Knowing the crucial role of macrophages and DCs in the activation of CD4 and CD8 T cells [73], it would be very interesting to define the role of MK produced by these APCs in their capacity to control T cells. Our results about the expression of MK in monocytes, macrophages and DCs in the tonsils suggest a role for MK in the ability of these cells to control B and T cells in the tonsils and other secondary lymphoid organs. For instance, tonsillar DCs are important for the stimulation of B cell responses [74], therefore it would be interesting to investigate whether MK that can influence the survival of B cells [1], as mentioned above, might participate in this process. Moreover, DCs and macrophages can influence the stimulation of T cells, and tonsillar DCs might have a regulatory or stimulatory effect on T cells depending on the cause of activation and the phenotype of the DCs [75, 76]. It is interesting then to understand the potential implication of MK in these effects; especially that MK has stimulatory effects on CD4 T cells [14], and either stimulatory or inhibitory effects on CD8 T cells [13, 15], as mentioned above. Moreover, DCs and macrophages might participate in the lymphangiogenesis that occurs in the lymph nodes that are enlarged during inflammation [24]. Taking into consideration our results about the capacity of DCs and macrophages to produce MK in the secondary lymphoid tissues, and the effect of MK on the proliferation of endothelial cells, it is important to assess the role of MK produced by DCs and macrophages in the lymphangiogenesis in these tissues.

MK plays a role in interfering with viral infections e.g. HIV [77] and in different physiological processes including reproduction, tissue repair, inflammation, innate immunity, control of blood pressure and angiogenesis [2]. It is also associated with diseases including rheumatoid arthritis, systemic lupus erythematosus, multiple sclerosis and cancers [2, 16–19]. Therefore, investigating the production of MK by innate APCs in these situations and aiming to regulate MK production by these cells might lead to beneficial outcomes including the inhibition of tumors growth. Moreover, MK implication in the resistance to anti-cancer therapy was shown [15]. MK is also able to regulate the activity of macrophages [13]. The role of MK produced by macrophages and DCs in these processes should be investigated.

## Conclusion

Altogether, MK is a cytokine that is produced by macrophages and MDDCs upon TLR ligation. NF-κB plays a potential role in the induction of MK production in macrophages and MDDCs upon TLR stimulation. Macrophages and MDDCs can stimulate endothelial cell proliferation through MK production. These results shed light on novel immunological phenomena that might have important therapeutic implications.
